# HLA and non-HLA genes and familial predisposition to autoimmune diseases in families with a child affected by type 1 diabetes

**DOI:** 10.1371/journal.pone.0188402

**Published:** 2017-11-28

**Authors:** Anna Parkkola, Antti-Pekka Laine, Markku Karhunen, Taina Härkönen, Samppa J. Ryhänen, Jorma Ilonen, Mikael Knip, the Finnish Pediatric Diabetes Register

**Affiliations:** 1 Scientific Laboratory, Children’s Hospital, University of Helsinki and Helsinki University Hospital, Helsinki, Finland; 2 Folkhälsan Research Center, Helsinki, Finland; 3 Research Programs Unit, Diabetes and Obesity, University of Helsinki, Helsinki, Finland; 4 Immunogenetics Laboratory, University of Turku, and Turku University Hospital, Turku, Finland; 5 Department of Political and Economic Studies, University of Helsinki, Helsinki, Finland; 6 Department of Pediatrics, Tampere University Hospital, Tampere, Finland; University of British Columbia, CANADA

## Abstract

Genetic predisposition could be assumed to be causing clustering of autoimmunity in individuals and families. We tested whether HLA and non-HLA loci associate with such clustering of autoimmunity. We included 1,745 children with type 1 diabetes from the Finnish Pediatric Diabetes Register. Data on personal or family history of autoimmune diseases were collected with a structured questionnaire and, for a subset, with a detailed search for celiac disease and autoimmune thyroid disease. Children with multiple autoimmune diseases or with multiple affected first- or second-degree relatives were identified. We analysed type 1 diabetes related HLA class II haplotypes and genotyped 41 single nucleotide polymorphisms (SNPs) outside the HLA region. The HLA-DR4-DQ8 haplotype was associated with having type 1 diabetes only whereas the HLA-DR3-DQ2 haplotype was more common in children with multiple autoimmune diseases. Children with multiple autoimmune diseases showed nominal association with *RGS1* (rs2816316), and children coming from an autoimmune family with rs11711054 (*CCR3-CCR5*). In multivariate analyses, the overall effect of non-HLA SNPs on both phenotypes was evident, associations with *RGS1* and *CCR3-CCR5* region were confirmed and additional associations were implicated: *NRP1*, *FUT2*, and *CD69* for children with multiple autoimmune diseases. In conclusion, HLA-DR3-DQ2 haplotype and some non-HLA SNPs contribute to the clustering of autoimmune diseases in children with type 1 diabetes and in their families.

## Introduction

With prevalence of up to 9%, autoimmune diseases (AID) constitute a significant disease burden [1]. In general, patients with these diseases and their relatives are at increased risk for other AIDs [1–3], although some diseases, for example multiple sclerosis and rheumatoid arthritis, are less likely to co-occur [1, 2, 4, 5]. Genetic predisposition to AIDs could be perceived as the reason for this clustering, although shared environmental factors might also play a role. Human leucocyte antigen (HLA) region on chromosome 6p21 mediates the strongest risk for many AIDs [6], but with modern genome-wide association analyses the number of genetic loci associated with autoimmunity has increased to more than one hundred. Most of these have functions related to different aspects of the immune system [7]. Accordingly, the genetic background for many AIDs is shared [7–10]; in a meta-analysis of genetics of pediatric AIDs, 81% of the associated loci were shared by at least two AIDs [11]. Many loci (e.g. *PTPN22*, *IL2RA*, *IL21* etc) have been implicated in multiple different AIDs, but the direction of effect is not always the same. For example, the A allele of the *PTPN22* SNP rs2476601 yields susceptibility to type 1 diabetes (T1D) and rheumatoid arthritis but protects from Crohn’s disease [11–13].

Type 1 diabetes (T1D) is an immune-mediated disease that affects the insulin producing β-cells and leads to lifelong dependency on exogenous insulin. The HLA region is estimated to explain about half of the genetic risk for T1D, and the number of other associated loci outside the HLA region is more than 50 to date [12,14–17]. Two HLA class II DR-DQ haplotypes, DRB1*03-DQA1*05-DQB1*02 (DR3-DQ2) and DRB1*0401/2/4/5-DQA1*0301-DQB1*0302 (DR4-DQ8), are associated with the risk of T1D, and especially DR3-DQ2 also with the risk of multiple other autoimmune diseases [6]. Some of the strongest risk genes for T1D outside the HLA region include the insulin gene (*INS*), *PTPN22*, and *IL2RA*. Out of these, *INS* is specific for T1D, whereas the others also convey risk for multiple other AIDs [11,18].

To test the hypothesis of an increased genetic risk load behind clustered autoimmunity we have recently compared children with T1D and a family history for T1D [19] or other AIDs [20] with children with no family history for AIDs. Children with familial T1D carried the DR4-DQ8 haplotype [19] and children with a family history for celiac disease the DR3-DQ2 [20] haplotype more frequently than children with sporadic T1D or no family history of AIDs, but taken together the differences were less conspicuous than expected. Moreover, the children with multiple AIDs did not differ from the children with T1D alone in terms of HLA class II genetics [20]. Therefore, as convincing evidence for an increased HLA class II mediated risk in families with clustered autoimmunity was lacking, we increased the sample size, and turned also to genes outside the HLA loci to test the hypotheses that HLA and non-HLA loci would explain the clustering of autoimmunity in children with multiple AIDs and contribute to the family history for AIDs.

## Materials and methods

### Patients

**Fig 1:**
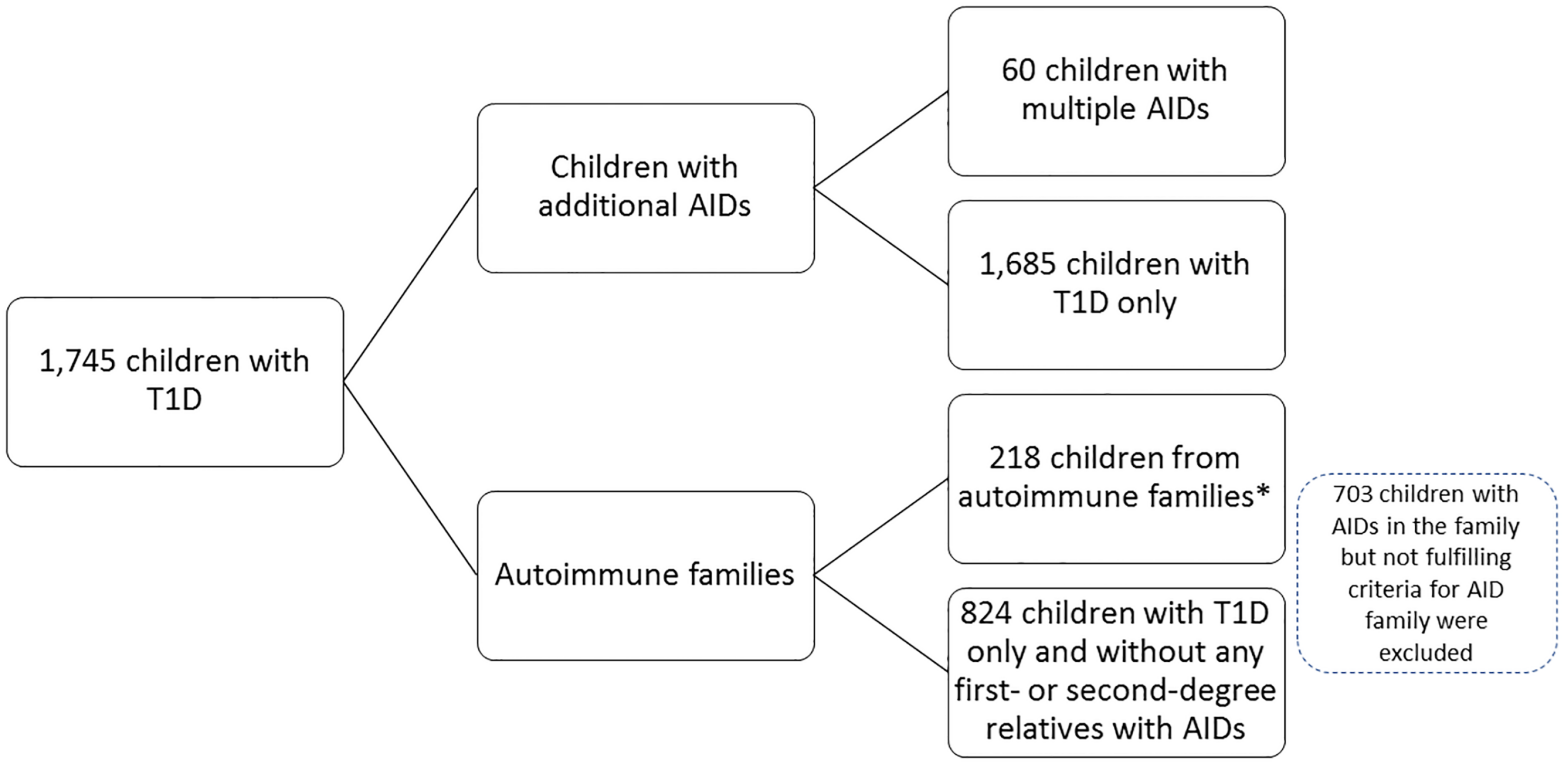
Groups of comparison used for the analyses. The children with multiple autoimmune diseases (AIDs) were compared with children with type 1 diabetes (T1D) only, and the children from autoimmune families to a group of children with T1D only and without any first- or second-degree relatives with any known AIDs. *Autoimmune families: ≥4 diagnoses of AIDs (T1D or other) and/or ≥3 different AIDs (T1D or other) in first- and/or second-degree relatives and/or the index child.

For the analysis of gene loci outside the HLA region, we included 1,784 children with T1D diagnosed before the age of 15 years (median 7.9 years, 57% male) from the Finnish Pediatric Diabetes Register. This is a nationwide register of children with newly diagnosed diabetes covering more than 90% of children diagnosed with T1D in the country since 2002. Data on family history of AIDs in first- and second-degree relatives at diagnosis of the index child was attained through questionnaires [19]. For a subset of cases, additional information on AIDs within the family was compiled when a new family member was diagnosed with diabetes and registered (n = 42), or when 40% of the children (n = 710) were included in a study with a search for autoimmune thyroid disease and screening for celiac autoimmunity [21]. For the analysis of phenotype-genotype associations, the cases were grouped into phenotype categories according to the history of AIDs (Fig 1): First, the children who themselves had another AID in addition to T1D, and second, the children coming from families with multiple AIDs (autoimmune families). A child was defined as belonging to an autoimmune family if there were more than three (≥4) diagnoses of AIDs (T1D or other) and/or more than two (≥3) different AIDs (T1D or other) in first- and/or second-degree relatives and/or the index child. The children with multiple AIDs (n = 60) were compared to children with T1D only (n = 1,685), and the children from autoimmune families (n = 218) to a group of children with T1D only and without any first- or second-degree relatives with any known AIDs (n = 824).

The ethics committee of Hospital District of Helsinki and Uusimaa has approved the protocol and all study participants and/or their legal guardians have signed an informed consent form. Participants over the age of 18 have signed an informed consent, and those over 10, an informed assent.

### Genotyping

The Sequenom (San Diego, California, USA) platform (Genome Center of Eastern Finland, University of Eastern Finland, Kuopio) was used to genotype 40 single nucleotide polymorphisms (SNPs) [22]. The chemokine (C-C motif) receptor 5 (*CCR5*) delta-32 deletion (rs333) was genotyped using fluorescent polymerase chain reaction primers and an automated sequencer (MegaBACE 1000, Amersham Biosciences, Buckinghamshire, UK) (Table 1). During quality control steps, 39 individuals failed the missingness tests of a genotyping call rate of at least 50% thus reducing the number of study subjects to 1,745. All the SNPs passed the genotyping call rate (at least 90%) and Hardy-Weinberg equilibrium tests (P > 0.001).

**Table 1:** The 41 single nucleotide polymorphisms (SNPs) genotyped in this study. The significant minor allele odds ratios (OR) for type 1 diabetes from the same population [22] are shown. To calculate combined numbers of risk alleles, two different calculations were used: one including only SNPs associated with AIDs other than type 1 diabetes (T1D), and another including also SNPs associated with T1D only (for autoimmune families). SNPs included in these two methods of calculations are marked with X in the two last columns. AA = Alopecia areata. AIT = autoimmune thyroid disease, CD = celiac disease, Crohn = Crohn’s disease, JIA = juvenile idiopathic arthritis, MAF = minor allele frequency in this dataset, OR = odds ratio for minor allele, PBC = primary biliary cirrhosis, PSO = psoriasis, RA = rheumatoid arthritis, SLE = systemic lupus erythematosus, T1D = type 1 diabetes, UC = ulcerative colitis, Vit = vitiligo. Site www.immunobase.org (accessed January 2017) was used for acquiring information on associations for this table.

									Combined number of risk alleles	
SNP	Gene	Minor/major allele	MAF	OR for T1D [22]	Risk allele in T1D	Risk allele in other autoimmune diseases	Associations with other autoimmune diseases for the locus	Reference for association in autoimmunity	Other autoimmune diseases	Autoimmune families
rs630115	*LOC646538*	A/G	0.33	0.88	G					x
rs2476601	*PTPN22*	A/G	0.22	1.79	A	A (G for Crohn)	AIT, Crohn, RA, SLE, Vit, JIA, AA	[28–34]	x	x
rs2816316	*RGS1*	G/T	0.13	0.83	T	T	CD	[15]	x	x
rs2984919	*RGS1*	A/T	0.13							
rs12061474	*PIK3C2B*	T/C	0.19		C					
rs3024505	*IL10*	T/C	0.14	0.87	C	T	Crohn, SLE, UC	[29, 32, 35]	x	x
rs6546909	*DQX1*	T/A	0.16		A					
rs9653442	*LOC150577/AFF3*	C/T	0.45		C	C	RA, JIA, CD	[36–38]	x	x
rs917997	*IL18RAP*	A/G	0.18		G	A	CD, Crohn, UC	[15, 32, 35]	x	x
rs2111485	*IFIH1*	A/G	0.38		G	G	PSO, Vit, SLE, UC, Crohn	[35, 39–41]	x	
rs1990760	*IFIH1*	C/T	0.37	0.85	T					x
rs7574865	*STAT4*	T/G	0.24	1.13	T	T	RA, SLE, PBC, CD, JIA	[29, 36, 42, 43]	x	x
rs3087243	*CTLA4*	A/G	0.31	0.84	G	G	AIT, CD, RA	[15, 28, 36]	x	x
rs11571297	*CTLA4*	G/A	0.34			G				
rs11711054	*CCR3-CCR5 region*	G/A	0.29		G	G	CD	[15, 24, 25]		
rs333	*CCR5-Delta32 delet*.	Del/-	0.13	0.78	No deletion	No deletion			x	x
rs17388568	*ADAD1*	A/G	0.42	1.20	A	A	JIA, UC	[33, 44]	x	x
rs7719828	*LOC645261*	T/C	0.29							
rs3757247	*BACH2*	A/G	0.38	1.15	A	A	Vit	[40]	x	x
rs6920220	*TNFAIP3*	A/G	0.21			A	RA,SLE, JIA, Crohn, UC	[16, 35, 45–47]	x	x
rs12722495	*IL2RA*	G/A	0.05	0.61	A		Vit, MS, RA	[16, 30, 48]		x
rs2104286	*IL2RA*	G/A	0.19	0.77	A	A			x	x
rs4749955	*IL2RA*	C/T	0.46							
rs11258747	*PRKCQ*	T/G	0.27		G		RA	[49]	x	x
rs2666236	*NRP1*	T/C	0.38		T					
rs689	*INS*	T/A	0.12	0.44	A					x
rs3764021	*CLEC2D*	G/A	0.48		G		MS	[50]	x	x
rs4763879	*CD69*	A/G	0.36		A					
rs1701704	*IKZF4*	C/A	0.37	1.31	C	C	Vit, AA	[40, 51]	x	x
rs4646536	*ERBB3*	A/C	0.34	1.25	A					
rs2292239	*CYP27B1*	C/T	0.34		T	T	MS, AA	[34, 50]	x	x
rs3184504	*SH2B3*	T/C	0.44	1.13	T	T	Vit, RA, CD, PBC, JIA, PSC	[37, 40, 42, 43, 52]	x	x
rs17696736	*C12orf30/NAA25*	G/A	0.42		G	G	JIA	[33]	x	x
rs9585056	*GPR183*	C/T	0.26		C	C	CD	[38]		
rs3825932	*CTSH*	T/C	0.37	0.85	C		CD, narcolepsy	[53, 54]	x	x
rs12708716	*CLEC16A/DEXI*	G/A	0.30	0.87	A	A	MS, PBC, CD	[24, 43, 50]	x	x
rs2903692	*CLEC16A*	A/G	0.28		G					
rs45450798	*PTPN2*	C/G	0.18	1.18	C		CD, Crohn, UC, RA, JIA	[15, 24, 33, 35, 42]	x	x
rs763361	*CD226*	T/C	0.46		T	T	MS, CD	[54, 55]	x	x
rs601338	*FUT2*	A/G	0.40	1.16	A	A	Crohn, PBC	[56, 57]	x	x
rs602662	*FUT2*	A/G	0.44		A	A				

To measure the combined load of risk alleles per individual, the absolute number of risk alleles per individual (0, 1, or 2 per SNP) was calculated. As we tested genetic associations with two different phenotypes, two different methods of calculating the combined number of risk alleles were used: For children with multiple AIDs, calculations included those loci reported to be associated with other AIDs (any AID, not just the particular disease the child has been diagnosed with) in the literature (Table 1). Only one SNP per locus was included, resulting in a total number of 25 SNPs for this calculation (Table 1). The allele associated with risk of autoimmunity was extracted from the literature (for *PTPN* SNP rs2476601 we used allele A which predisposes to T1D, rheumatic arthritis, systemic lupus erythematosus, and vitiligo but protects from Crohn’s disease). If such a risk allele could not be named unambiguously from previous studies (four SNPs), we assumed the risk allele for autoimmunity to be the same as that for T1D. For autoimmune families, which include clusters of both T1D and other AIDs, we included the SNPs associated with other AIDs as well as those associated with T1D in a previous study of the same population [18] [28 SNPs, Table 1, rs2292239 (*ERBB*) was excluded due to its strong linkage disequilibrium with rs1701704 (*IKZF4*)]. Only the children who had results available for all 25 (n = 1,471) or 28 (n = 1,457) SNPs were included in the calculations.

### Statistical analyses

#### Univariate analyses

Association analyses for non-HLA SNPs were carried out with the PLINK v1.07 software package (http://pngu.mgh.harvard.edu/purcell/plink/), and analyses for HLA haplotypes/genotypes and the number of risk alleles with IBM SPSS Statistics 24. Absolute combined numbers of risk alleles were analyzed with Student’s t test and HLA prevalences with cross tabulation/χ2-statistics with Yates continuity correction or Fisher’s exact test when appropriate. A two-sided P value of <0.05 was considered nominally significant. For the association analyses the P values were corrected for multiple comparisons by the false discovery rate (FDR) step-up procedure by Benjamini and Hochberg in PLINK. Power calculations were performed with the Quanto 1.2.4 (http://hydra.usc.edu/gxe, 2006) software. In the univariate analyses, assuming log-additive mode of inheritance, two-sided type I error of 0.05, and minor allele frequencies (MAF) from 0.05 to 0.48, our study had 80% power to detect ORs of 2.6 (MAF 0.05) to 1.7 (MAF 0.45) for the phenotype of children with multiple AIDs and an OR of 1.9 to 1.4 for the phenotype of autoimmune families.

#### Multivariate analyses

In genome-wide association studies, the number of SNPs typically exceeds the number of samples by orders of magnitude. In these data, the number of SNPs (41) and other covariates (13; age, sex, clinical covariates, β-cell autoantibodies, and HLA; a total of 54 covariates) was lower than the number of children with complete information on all the 54 variables (e.g.1,427 for the HLA analysis, 1,180 for the non-HLA SNPs for the phenotype of children with multiple AIDs). Consequently, it was possible to build standard regression models to analyze the joint explanatory power of the genomic loci. Accordingly, we built four multivariate models to answer the following questions: Do HLA class II haplotypes help to predict additional AID in a child with T1D (model 1) or the AID status of the family (autoimmune families, model 2), and do the non-HLA SNPs help to predict additional AID in a child with T1D (model 3) or the AID status of the family (model 4)?

We modelled the additional AID of the child (models 1 and 3) and the AID status of the family (models 2 and 4) as a binary phenotype, i.e. we used the same phenotype as in the univariate analyses. We applied generalized linear models with a complementary log-log link function to estimate the parameters (for derivation, see Supporting information). In models 3 and 4, we left out eight SNPs that were in linkage disequilibrium with other SNPs (rs2984919, rs2111485, rs11571297, rs12722495, rs4749955, rs2292239, rs2903692 and rs602662). This was done to avoid numerical artefacts arising from multicollinearity.

In each model, we included a set of potential confounding factors: sex, body mass index (BMI), plasma glucose levels at diagnosis, blood pH at diagnosis, log-levels of β-cell autoantibodies at diagnosis [autoantibodies against glutamic acid decarboxylase (GADA), autoantibodies against the IA-2 molecule (IA-2A), insulin autoantibodies (IAA), islet autoantibodies (ICA), autoantibodies against the zinc transporter 8 (ZnT8A)], and for models 3 and 4, the HLA risk group coded as a continuous variable. The HLA risk group is a classification for HLA class II mediated risk for T1D with six groups from protective (risk group 0) to strongly increased risk (risk group 5) [23]. (We also tried coding the HLA risk group as a categorical variable, but there were not enough samples to identify the coefficients.) In models 1 and 3, we included the log-age of the child as an offset, whereas in models 2 and 3, we included log-number of family members as an offset (for justification, see Supporting information).

We performed model choice over the SNP covariates (i.e. HLA class II haplotypes in models 1 and 2, and the 33 non-linked, non-HLA SNPs in models 3 and 4). We used stepwise forward model selection to minimize Akaike’s information criterion (AIC). Thus, the starting point is that the outcome of interest is affected only by the confounding factors, which are always included in the model, and the SNP covariates are added only if they improve the information criterion. After the model choice, we measured the joint explanatory power of the SNP covariates by comparing the chosen model with the minimal model (only confounding factors) by using the likelihood-ratio test statistic.

We performed all calculations in R, using the standard functions glm and step for estimation and model choice, respectively. We also run a series of alternative models to check the robustness of results towards model specifications (see Supporting information). More specifically, we tested the robustness of results towards the model choice algorithm [forward selection as opposed to backward selection, AIC as opposed to Bayesian information criterion (BIC)], and the treatment of person-time at risk.

## Results

### Univariate analyses

Only 27 of the 1,745 children had another AID diagnosed already at diagnosis of T1D but 33 additional children were diagnosed later resulting in a total number of 60 cases (3.4%) with this phenotype. The other AIDs diagnosed in these children were celiac disease (n = 29), autoimmune thyroid disease (n = 24), rheumatoid arthritis (n = 4), vitiligo (n = 2), alopecia (n = 2), and colitis ulcerosa (n = 1). One child had both celiac disease and autoimmune thyroid disease and another had both celiac disease and vitiligo.

Compared to children with T1D only, the children with multiple AIDs carried less often the HLA-DR4-DQ8 haplotype (70.8 vs. 50.0%, P = 0.001) and the DR4-DQ8/y genotype (y ≠ DR3-DQ2, 49.7 vs. 35.0%, P = 0.04), whereas the DR3-DQ2/x genotype (x ≠ DR4-DQ8) was common among children with multiple AIDs (31.7 vs. 13.7%, P < 0.001) (Table 2). Also, the risk group distribution varied between the groups; the children with T1D only were characterized by high prevalence of the highest risk group, and the children with multiple AIDs with a low risk group (risk group 1 especially) (Table 2). A total of 218 children (12.5%) belonged to autoimmune families. For this phenotype comparison, there were no differences in HLA profiles (Table 2).

**Table 2:** HLA haplotypes and genotypes in clustering autoimmunity. HLA class II risk groups, haplotypes and genotypes were compared between children with multiple autoimmune diseases and those with T1D only, as well as children from autoimmune families and children without any additional autoimmune diseases in the family. (Autoimmune families: ≥4 diagnoses of autoimmune diseases (T1D or other) and/or ≥3 different autoimmune diseases (T1D or other) in first- and/or second-degree relatives and/or the index child). For the comparison of risk groups, a joint P value is shown. Five children did not have any information on their HLA genetics and were thus excluded from this analysis; none of the five had other autoimmune diseases and one came from an autoimmune family.

HLA, %	Children with multiple autoimmune diseases (n = 60)		Children with T1D only (n = 1,680)		P value	Children from autoimmune families (n = 217)		Children without family history of autoimmune diseases (n = 820)		P value
	n	%	n	%		n	%	n	%	
Risk group										
0	0	0	17	1.0	**0.02**	0	0	9	1.1	0.56
1	6	10.0	33	2.0		6	2.9	18	2.2	
2	6	10.0	247	14.7		34	15.7	124	15.2	
3	14	23.3	390	23.3		49	22.6	210	25.7	
4	25	41.7	636	37.9		80	36.9	299	36.6	
5	9	15.0	353	21.1		48	21.1	156	19.1	
DR3-DQ2/DR4-DQ8	9	15.0	354	21.1	0.27	48	22.1	156	19.1	0.39
DR3-DQ2/x^a^	19	31.7	230	13.7	<**0.001**	32	14.7	124	15.2	0.92
DR4-DQ8/y^b^	21	35.0	832	49.7	**0.04**	104	47.9	400	49.0	0.82
x^a^/y^b^	11	18.3	259	15.5	0.59	33	15.2	136	16.6	0.68
DR4-DQ8 haplotype	30	50.0	1188	70.8	**0.001**	152	70.0	559	68.3	0.62
DR3-DQ2 haplotype	28	46.7	583	34.8	0.07	80	36.9	280	34.2	0.47

^a^ x ≠ DR4-DQ8

^b^ y ≠ DR3-DQ2

With univariate association analysis, compared to children with type 1 diabetes only (n = 1,685), the phenotype of having multiple AIDs associated nominally with two *RGS1* SNPs (Table 3); rs2816316 (OR 1.88, P = 0.006) and rs2984919 (OR 1.82, P = 0.008). Belonging to an autoimmune family associated with rs11711054 (*CCR3-CCR5*, OR 0.69, P = 0.003). Correcting for multiple tests in the univariate association analyses removed statistical significance from these findings, however (Table 3).

**Table 3:** Results of association analyses for 41 single nucleotide polymorphisms (SNPs) analysed. Odds ratios (OR) with 95% confidence intervals (CI) for the minor alleles of these SNPs for children with T1D with two different phenotypes of clustered autoimmunity are shown. Children with multiple autoimmune diseases were compared to those with T1D only (n = 1,685) and children from autoimmune families to children without any additional autoimmune diseases in the family (n = 824). Autoimmune families: ≥4 diagnoses of autoimmune diseases (T1D or other) and/or ≥3 different autoimmune diseases (T1D or other) in first- and/or second-degree relatives and/or the index child. (CHR = chromosome, MAF = minor allele frequency, FDR P value = false discovery rate corrected P value).

CHR	SNP	Gene	Minor/major allele	MAF	Children with multiple autoimmune diseases (n = 60)				Children from autoimmune families (n = 218)			
					OR	95% CI	P value	FDR P value	OR	95% CI	P value	FDR P value
1	rs630115	*LOC646538*	A/G	0.33	1.20	0.82–1.76	0.35		0.93	0.74–1.17	0.53	
1	rs2476601	*PTPN22*	A/G	0.22	0.97	0.62–1.51	0.89		0.94	0.72–1.23	0.67	
1	rs2816316	*RGS1*	G/T	0.13	**1.88**	**1.20–2.96**	**0.006**	0.17	1.06	0.77–1.44	0.73	
1	rs2984919	*RGS1*	A/T	0.13	**1.82**	**1.16–2.86**	**0.008**	0.17	1.05	0.77–1.43	0.74	
1	rs12061474	*PIK3C2B*	T/C	0.19	1.05	0.66–1.67	0.83		0.99	0.75–1.30	0.94	
1	rs3024505	*IL10*	T/C	0.14	0.63	0.34–1.19	0.15		0.91	0.66–1.25	0.57	
2	rs6546909	*DQX1*	T/A	0.16	0.89	0.53–1.51	0.68		0.86	0.64–1.16	0.32	
2	rs9653442	*LOC150577*	C/T	0.45	0.88	0.61–1.28	0.52		0.99	0.80–1.23	0.92	
2	rs917997	*IL18RAP*	A/G	0.18	0.73	0.44–1.23	0.24		0.97	0.73–1.28	0.81	
2	rs2111485	*IFIH1*	A/G	0.38	0.90	0.61–1.32	0.59		0.89	0.71–1.11	0.31	
2	rs1990760	*IFIH1*	C/T	0.37	0.95	0.64–1.39	0.78		0.92	0.73–1.14	0.43	
2	rs7574865	*STAT4*	T/G	0.24	0.98	0.64–1.51	0.93		1.00	0.78–1.29	0.99	
2	rs3087243	*CTLA4*	A/G	0.31	0.82	0.55–1.24	0.35		0.98	0.78–1.24	0.89	
2	rs11571297	*CTLA4*	G/A	0.34	0.78	0.53–1.17	0.23		0.97	0.78–1.22	0.81	
3	rs11711054	*CCR3-CCR5*	G/A	0.29	0.98	0.65–1.47	0.91		**0.69**	**0.54–0.88**	**0.003**	0.13
3	rs333	*CCR5-Delta32 delet*.	T/A	0.13	1.00	0.56–1.76	0.99		1.06	0.78–1.45	0.70	
4	rs17388568	*ADAD1*	A/G	0.42	1.35	0.94–1.95	0.11		0.95	0.77–1.18	0.66	
5	rs7719828	*LOC645261*	T/C	0.29	1.10	0.74–1.64	0.63		1.17	0.93–1.47	0.18	
6	rs3757247	*BACH2*	A/G	0.38	1.07	0.74–1.55	0.72		0.97	0.78–1.20	0.76	
6	rs6920220	*TNFAIP3*	A/G	0.21	1.42	0.94–2.14	0.097		1.23	0.95–1.59	0.11	
10	rs12722495	*IL2RA*	G/A	0.05	0.64	0.23–1.76	0.38		1.18	0.76–1.84	0.47	
10	rs2104286	*IL2RA*	G/A	0.19	0.96	0.60–1.56	0.88		1.11	0.86–1.44	0.43	
10	rs4749955	*IL2RA*	C/T	0.46	0.87	0.60–1.25	0.44		1.05	0.85–1.30	0.65	
10	rs11258747	*PRKCQ*	T/G	0.27	1.22	0.82–1.81	0.33		0.97	0.76–1.24	0.82	
10	rs2666236	*NRP1*	T/C	0.38	0.75	0.50–1.13	0.17		1.03	0.83–1.29	0.78	
11	rs689	*INS*	T/A	0.12	1.21	0.71–2.08	0.48		0.86	0.61–1.22	0.40	
12	rs3764021	*CLEC2D*	G/A	0.48	1.42	0.98–2.05	0.06		1.02	0.82–1.26	0.87	
12	rs4763879	*CD69*	A/G	0.36	1.45	1.00–2.09	0.05	0.66	0.99	0.79–1.24	0.94	
12	rs1701704	*IKZF4*	C/A	0.37	1.05	0.72–1.54	0.79		1.20	0.97–1.50	0.10	
12	rs4646536	*CYP27B1*	C/T	0.34	1.32	0.90–1.91	0.15		0.92	0.73–1.15	0.47	
12	rs2292239	*ERBB3*	A/C	0.34	1.00	0.67–1.50	0.99		1.23	0.98–1.54	0.07	
12	rs3184504	*SH2B3*	T/C	0.44	1.01	0.69–1.46	0.98		1.10	0.89–1.36	0.39	
12	rs17696736	*C12orf30 = NAA25*	G/A	0.42	0.96	0.65–1.42	0.84		1.01	0.81–1.26	0.94	
13	rs9585056	*GPR183*	C/T	0.26	0.85	0.55–1.31	0.46		1.21	0.96–1.54	0.11	
15	rs3825932	*CTSH*	T/C	0.37	0.88	0.60–1.29	0.50		1.04	0.84–1.30	0.71	
16	rs12708716	*CLEC16A*	G/A	0.30	1.22	0.82–1.80	0.33		1.09	0.86–1.37	0.49	
16	rs2903692	*CLEC16A*	A/G	0.28	1.19	0.80–1.77	0.39		1.06	0.83–1.34	0.66	
18	rs45450798	*PTPN2*	C/G	0.18	1.06	0.67–1.69	0.79		0.87	0.65–1.15	0.33	
18	rs763361	*CD226*	T/C	0.46	1.18	0.82–1.69	0.38		1.04	0.84–1.28	0.74	
19	rs601338	*FUT2*	A/G	0.40	1.27	0.88–1.82	0.21		0.92	0.74–1.15	0.45	
19	rs602662	*FUT2*	A/G	0.44	1.24	0.86–1.79	0.26		0.84	0.68–1.05	0.12	

**Fig 2:**
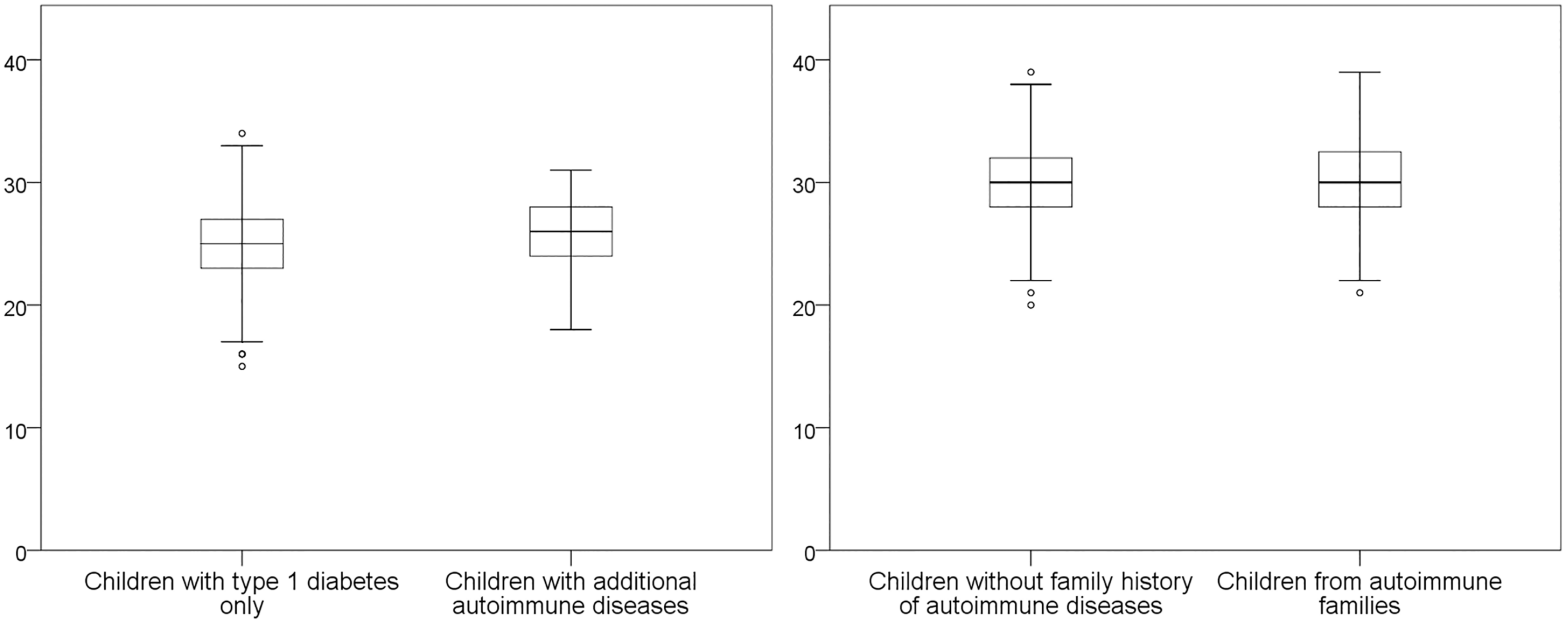
Combined mean number of risk alleles per subject compared between the groups. Mean number of risk alleles (0, 1, or 2 per SNP) were compared between children with type 1 diabetes only and children with multiple autoimmune diseases left, P = 0.39), and children without any family history of autoimmune disease and children from autoimmune families (≥4 diagnoses of autoimmune diseases and/or ≥3 different autoimmune diseases in the extended family, right, P = 0.38).

For the comparison of combined number of risk alleles, 25 SNPs were included for children with multiple AIDs. The mean was 25.2 (range 15–24) of the theoretical maximum of 50 risk alleles. For the combination of both T1D and other AID SNPs (28 SNPs), the mean number of risk alleles was 30.2 (range 18–39) of the maximum of 56. These calculated mean numbers of risk alleles did not differ significantly between either pair of the phenotypes (Fig 2).

### Multivariate analyses

By comparing the model with only potential confounding factors and another with HLA haplotypes, we concluded that a model including DR4-DQ8 was stronger in explaining multiple AIDs in children with T1D (Model 1: AIC 470 vs. 462, P = 0.0019 from the LR test). Specifically, carrying the DR4-DQ8 haplotype yielded reduced risk for having multiple AIDs (Table 4). After the model selection, the final model did not include the DR3-DQ2 haplotype. Therefore, the joint P value measures the effect of DR4-DQ8 alone. In contrast, the HLA haplotypes did not improve the model fit for explaining autoimmune families (only confounding factors are retained in the model, Table 4). Lower plasma glucose level of the index child at diagnosis associates with belonging to an autoimmune family.

**Table 4:** HLA class II haplotypes in prediction of clustering of autoimmune diseases. The results of multivariate models 1 and 2 with research questions: Do HLA class II haplotypes help to predict clustering of autoimmune diseases in children with multiple autoimmune diseases (Model 1), or in children from autoimmune families (Model 2). For Model 1, the statistics are: AIC for the confounding factors alone 470, AIC of this model 462, joint P value 0.0019. The joint P value concerns the effect of HLA class II genotypes. After model selection, Model 2 included only the confounding factors. HR = hazard ratio for the minor allele, CI = Confidence interval. Only children with information available on all covariates and were included in the analyses.

Covariate	HR	95% CI	P value (Wald’s test)
Model 1. Children with multiple autoimmune diseases, total n = 1,427			
Female sex	1.57	0.91–2.73	0.11
Body mass index (BMI)	0.91	0.80–1.01	0.084
Plasma glucose at diagnosis	0.99	0.96–1.01	0.34
Blood pH at diagnosis	4.19	0.21–149.7	0.39
log-GADA level	1.02	0.87–1.21	0.81
log-IA2A level	0.93	0.80–1.10	0.42
log-IAA level	1.09	0.88–1.32	0.41
log-ICA level	0.95	0.77–1.19	0.67
log-ZnT8A level	0.84	0.65–1.08	0.19
DR4-DQ8	**0.41**	**0.23–0.72**	**0.0019**
Model 2. Autoimmune families, total n = 842			
Female sex	1.23	0.91–1.66	0.17
Body mass index (BMI)	1.03	0.97–1.08	0.33
Plasma glucose at diagnosis	**0.98**	**0.96–1.00**	**0.023**
Blood pH at diagnosis	3.26	0.66–19.02	0.17
log-GADA level	1.08	0.98–1.18	0.10
log-IA2A level	1.03	0.94–1.12	0.58
log-IAA level	1.04	0.94–1.16	0.42
log-ICA level	0.96	0.85–1.09	0.56
log-ZnT8A level	0.91	0.80–1.04	0.19

Analyzing the contribution of non-HLA SNPs, the model with SNPs was superior to the basic model for both phenotypes (AIC 365 vs. 382, P < 0.0001 for model 3 and AIC 710 vs. 716, P = 0.006 for model 4, Table 5). The specific SNPs associated with having multiple AIDs were neuropilin 1 (*NRP1*, rs2666236), *RGS1* (rs2816316), *CD69* (rs4763879), and *FUT2* (601338, Table 5). The direction of effect for *RGS1* association was the same as in the univariate analyses (minor allele increases risk for other AIDs). The specific SNP associated with autoimmune families was *CCR3-CCR5* region (rs11711054, Table 5), and the direction of effect was the same as in the univariate analysis (minor allele protective). Other significantly associated variables were lower plasma glucose and higher GADA titers for autoimmune families.

**Table 5:** Non-HLA SNPs in prediction of clustering of autoimmune diseases. The results of multivariate models 3 and 4 with research questions: Do the 33 non-linked, non-HLA SNPs (single nucleotide polymorphisms) help to predict clustering of autoimmune diseases in children with multiple autoimmune diseases (Model 3), or in children from autoimmune families (Model 4). For Model 3, the statistics are: AIC for the confounding factors alone 382, AIC of this model 365, joint P value 6.6×10^−5^. For Model 4, the statistics are: AIC for the confounding factors alone 716, AIC of this model 710, joint P value 0.0059. The joint P values concern the effects of the non-HLA SNPs. HR = Hazard ratio for the minor allele, CI = Confidence interval. Only children with information available on all variables were included in the analyses.

Covariate	HR	95% CI		P value (Wald’s test)
Model 3. Children with multiple autoimmune diseases, total n = 1,180				
Female sex	1.73	0.94–3.23		0.082
Body mass index (BMI)	0.92	0.81–1.04		0.20
Plasma glucose at diagnosis	0.96	0.93–1.00		0.052
Blood pH at diagnosis	1.77	0.08–73.78		0.74
log-GADA level	0.93	0.78–1.12		0.46
log-IA2A level	0.87	0.73–1.05		0.13
log-IAA level	1.06	0.83–1.32		0.63
log-ICA level	1.00	0.78–1.29		0.98
log-ZnT8A level	0.84	0.62–1.11		0.24
HLA risk level	0.78	0.59–1.02		0.065
rs2666236 (*NRP1*)	**0.52**	**0.31–0.83**		**0.0081**
rs763361 (*CD226*)	1.38	0.89–2.13		0.15
rs2816316 (*RGS1*)	**2.17**	**1.20–3.8**		**0.0071**
rs601338 (*FUT2*)	**1.58**	**1.05–2.40**		**0.028**
rs3024505 (*IL10*)	0.51	0.21–1.04		0.093
rs4763879 (*CD69*)	**1.67**	**1.09–2.55**		**0.019**
Model 4. Autoimmune families, total n = 691				
Female sex	1.21	0.87–1.68		0.26
Body mass index (BMI)	1.02	0.96–1.09		0.43
Plasma glucose at diagnosis	**0.97**	**0.96–0.99**		**0.006**
Blood pH at diagnosis	1.6	0.29–10.62		0.61
log-GADA level	**1.11**	**1.00–1.22**		**0.049**
log-IA2A level	1.01	0.92–1.12		0.84
log-IAA level	1.02	0.91–1.14		0.72
log-ICA level	0.93	0.80–1.07		0.28
log-ZnT8A level	0.94	0.82–1.08		0.40
HLA risk level	1.04	0.89–1.21		0.64
rs6546909 (*DQX1*)	0.75	0.53–1.05		0.11
rs11711054 (*CCR3-CCR5*)	**0.70**	**0.53–0.91**		**0.01**
rs7719828 (*LOC645261*)	1.21	0.95–1.53		0.12
rs9585056 (*GPR183*)	1.2	0.94–1.53		0.13

These reported results of the multivariate analyses are largely robust towards alternative model specifications (Supporting information). Robustness checks were performed with backward instead of forward model selection, with models minimizing BIC instead of AIC, and with alternative person-time. Specifically, the associations of DR4-DQ8 with autoimmune clustering within the child, and rs11711054 (*CCR3-CCR5*) with the familial phenotype are seen in all alternative models, and with the same direction of effect. The association between *RGS1* and the additional AID within the child is found in two of the alternative model specifications (backward model selection and alternative person time, see Supporting information), whereas BIC tends to favour a model without *RGS1*.

## Discussion

We aimed at identifying genetic risk loci for clustering of AIDs. A reasonable hypothesis is that the genetic risk for autoimmunity is greater in individuals and families with multiple diagnoses of AIDs, and that for example the SNPs strongly associated with multiple AIDs (such as *IL2RA* or *PTPN22* [11, 18]) would be associated with clustering of these diseases. In the association analyses, some genetic loci reached nominally significant association with clustered AIDs but these findings did not survive correction for multiple testing. Multivariate analyses, however, indicated significant contributions of non-HLA SNPs to the clustering of AIDs. The effects of non-HLA SNPs were independent of both the HLA risk group and potential confounding factors. Moreover, in the multivariate analyses, associations from the univariate analyses were repeated, namely for children with multiple AIDs one of the *RGS1* SNPs (rs2816316) and, for autoimmune families, rs11711054 intergenic between *CCR3* and *CCR2* region. Also, *NRP1*, *FUT2*, and *CD69* associated with having other AIDs in addition to T1D.

The gene product of *RGS1* is a regulator of G-protein signaling implicated in the activation and proliferation of B cells. According to our results the minor G allele of rs2816316 (*RGS1)* confers risk for having multiple AIDs, whereas in previous reports it has been the major T allele that predisposes to T1D and celiac disease [15, 22]. However, the risk of multiple AIDs was tested against a group of children with T1D only, not against control subjects. Thus, the increase of an allele not associated with T1D may just reflect decreased risk for T1D. A similar phenomenon was also observed when a decreased risk of having multiple AIDs was associated with HLA DR4-DQ8 –the haplotype conferring the highest risk for T1D.

Similarly, the T1D and celiac disease associated minor allele G of rs11711054 (*CCR3-CCR5*) was found to be decreased in children from autoimmune families. The earlier detected association with T1D is weak and not at all detected in our Finnish family series [22]. In contrast, the reported association with celiac disease is stronger [24, 25], which is difficult to link to our finding of a decreased frequency in children from autoimmune families. This SNP is located in the chemokine receptor gene cluster region containing the *CCR3*, *CCR2*, and *CCR5* genes. These proteins are involved in multiple aspects of the immune system, and the CCR2 molecule has been shown to be downregulated in dendritic cells of T1D patients [26].

Accordingly, these loci are credible risk loci for clustered autoimmunity, as they have functions in the immune system. Interestingly, the SNPs previously reported to be associated with multiple pediatric AIDs, for example *PTPN22* and *IL2RA* [11], did not reach significance in our study, which is difficult to explain. However, our selection of SNPs is more limited and different from other similar studies, making comparisons difficult.

In autoimmune families, HLA did not play a significant role, but in children with multiple AIDs, it showed a significant contribution. Namely, DR3-DQ2 and especially the DR3-DQ2/x genotype, associated with having multiple autoimmune diseases, and the children with T1D only were characterized by having DR4-DQ8 (Table 2). The same was seen in the multivariate analysis with the DR4-DQ8 haplotype protecting from having multiple AIDs (Table 3). This is understandable, as this is the haplotype with the strongest predisposition to T1D and the prevalence in our study population was 70%. The children with multiple AIDs carried the HLA risk group 1, which is associated with decreased risk for type 1 diabetes, conspicuously often. In fact, 11% of the children with a decreased risk group (risk groups 0 and 1), compared to 3% of children from other risk groups had other AIDs in addition to T1D. Accordingly, the children who develop T1D despite of a protective HLA class II genotype, seem to have a propensity to a broader range of AIDs. This patient group represents an interesting subgroup for future studies on clustering of autoimmunity in T1D.

As older age at diagnosis associated with risk of multiple AIDs (these children have had more time to be diagnosed with another disease) in our and others’ datasets [27], age at diagnosis was included in the multivariate analyses as an offset. Higher GADA titers and lower plasma glucose levels were characteristic of children from autoimmune families. GADA are associated with a general propensity to autoimmunity [27] while lower glucose levels in these children can be explained by earlier recognition of the disease in families with previous experience of T1D [19].

Our sample size was small, despite being based on a large nationwide register, for association analyses. Therefore, we used additional multivariate methods to provide more evidence for the involvement of SNPs outside the HLA region in clustering of AIDs; the models including the SNPs were superior to basic models by P-values of 3.9×10^−5^ and 0.006 in models 3 and 4, respectively. Although these joint P-values cannot be directly used to partition this association to individual SNPs, some indication is provided by the individual HRs and P values (Table 4). To increase power to our analysis, we included also T1D to criteria used to define autoimmune family making the number of autoimmune families 218. If only families with a history of AIDs other than T1D would have been considered, the number of cases would have been significantly smaller (n = 83) and the comparison would have been limited to AIDs other than T1D. The current analysis includes also familial T1D. The multivariate analyses were as well limited to cases with data available on all included variables reducing the total number of included cases from 1,745 to 1,427 and 1,180 for models one and three, respectively (children with multiple AIDs), and from 1,042 to 842 and 691 for models two and four, respectively (children from autoimmune families). This reduces power of these analyses but, in our view, is unlikely to bias the results significantly.

Another potential limitation of our study is the selection of SNPs, which were primarily chosen for risk association with T1D. Thus, for the phenotypes for clustering of heterogeneous other AIDs, a different set of SNPs might have been more appropriate, although majority of the 41 SNPs tested are associated also with other AIDs (Table 1). We therefore cannot rule out the possibility that major non-HLA SNPs not associated with T1D, but predisposing children with T1D to other AIDs, do exist. A study with a larger set of SNPs could clarify this issue. Also, we grouped the heterogeneous group of AIDs together when in fact some of the genetic risk factors are likely disease specific. The ascertainment of the phenotypes of autoimmunity mainly by self-reported questionnaires and at one time-point could also affect our findings. Our data represents mainly the family history of AIDs at diagnosis of T1D of the index case and only a subset of the cases was systematically searched for celiac disease and autoimmune thyroiditis later. However, a more detailed search for autoimmune family history would have led to an even smaller sample size. We are also unable to provide independent validation of our findings in a second cohort.

Our study represents an effort to use the ample findings of genome-wide association studies in subtyping T1D. In conclusion, non-HLA SNPs—and also HLA for the children with multiple AIDs—contribute to the clustering of autoimmunity. We confirmed the association of HLA-DR3-DQ2 positive T1D with other autoimmune diseases and discovered credible candidate loci for clustering of AIDs: *RGS1* and possibly *NRP1*, *FUT2*, and *CD69* are risk loci for having other AIDs in addition to T1D, and within the region between *CCR3* and *CCR5* might exist a risk locus for clustering of AIDs in families of patients affected by T1D. The overall number of risk alleles did not differ between the groups, however. Nevertheless, also the environmental factors shared by family members, such as the microbial diversity and hygiene level of the environment and the intestinal microflora, most likely contribute to the familial clustering of AIDs.

## Supporting information

S1 File Supplemental methods. Deriving the multivariate models and robustness checks.
(PDF)

S2 File Phenotype and genotype data of the subjects. (XLSX)
