# Supplementary material for: HLA and non-HLA genes and familial predisposition to autoimmune diseases in families with a child affected by type 1 diabetes
Source: PLoS One. 2017 Nov 28;12(11):e0188402. doi: 10.1371/journal.pone.0188402 (PMC5705143; doi:10.1371/journal.pone.0188402)
Supplement: S1 File — Deriving the multivariate models and robustness checks. (PDF) [file pone.0188402.s001.pdf]

# Supporting information

Supplement to: Parkkola A, Laine AP, Karhunen M, Härkönen T, Ryhänen SJ, Ilonen J, Knip M, and the Finnish Pediatric Diabetes Register. Non-HLA genes and familial predisposition to autoimmune diseases in families with a child affected by type 1 diabetes. *PLOS ONE.*

# Supplemental methods

## Deriving the multivariate models

We use generalized linear models (e.g. McCullagh and Nelder 1989) to investigate the relationship between binary outcomes and different covariates of interest. These models are typically used as ad-hoc tools, merely by assuming some link function (e.g. logit regression). In this section we show, how a complementary log-log link function arises naturally from assuming proportional hazards between different genotypes.

In models 1 and 3, we investigate, whether the SNP covariates (HLA class II haplotypes in model 1, and non-HLA SNPs in model 3) help to predict additional autoimmune diseases (AIDs) in a child. Let us assume that the baseline risk of contracting any non-diabetic AID is a constant $\lambda_0$ per time unit. Then it follows that the probability of having at least one non-diabetic AID at age $t_i$ is

$$F(t_i) = 1 - S(t_i) = 1 - e^{-\lambda_0 t_i}. \quad \text{(A1)}$$

Now, let us consider individual $i$ with individual-specific covariates $\boldsymbol{x}_i$ and age at diagnosis $t_i$. If we assume proportional hazards, the individual-specific risk is given by

$$\lambda_i = \lambda_0 e^{\boldsymbol{x}_i' \boldsymbol{\beta}} \quad \text{(A2)}$$

where $\boldsymbol{\beta}$ is a vector of regression coefficients. It follows from (A2) that the individual-specific probability is given by

$$F_i(t_i) = 1 - \exp\{-\lambda_i t_i\} = 1 - \exp\left\{-\lambda_0 e^{\boldsymbol{x}_i' \boldsymbol{\beta}} t_i\right\}, \quad \text{(A3)}$$

and consequently,

$$\log\{-\log[1 - F_i(t_i)]\} = \log \lambda_0 + \boldsymbol{x}_i' \boldsymbol{\beta} + \log t_i. \quad \text{(A4)}$$

Equation A4 can be estimated by using a generalized linear model with a complementary log-log link for binary data. The age at diagnosis is to be modelled as an offset. However, we also estimate an alternative model

$$\log\{-\log[1 - F_i(t_i)]\} = \log \lambda_0 + \boldsymbol{x}_i' \boldsymbol{\beta} + \gamma \log t_i \quad \text{(A5)}$$

as a robustness check. In this model, $\gamma$ is a parameter to be estimated freely, and consequently, log-age is treated as any other covariate.

In models 2 and 4, we investigate, whether the SNP covariates (HLA class II haplotypes in model 2, and non-HLA SNPs in model 4) help to predict the AID status of a family. If we assume proportional hazards in line with A1, then the joint hazard of the family $i$ can be modelled as

$$\lambda_i = \lambda_0 \sum_{j=1}^{n_i} e^{\boldsymbol{x}_{ij}' \boldsymbol{\beta}} \quad \text{(A6)}$$

where $\boldsymbol{x}_{ij}$ denotes the vector of covariates for the $j$th member of family $i$, and $n_i$ denotes the size of family $i$. However, we do not observe $\boldsymbol{x}_{ij}$ directly, and $n_i$ changes in time in an unobserved manner. Thus, we assume that the family-specific risk depends on the covariates of the index patient (denoted by $\boldsymbol{x}_i$) and the number of family members as

$$\lambda_i = \lambda_0 e^{\boldsymbol{x}_i' \boldsymbol{\beta}} n_i. \quad \text{(A7)}$$

In line with A3 and A4, we derive the model to be estimated as

$$\log\{-\log[1 - F_i(t_i)]\} = \log \lambda_0 + \boldsymbol{x}_i'\boldsymbol{\beta} + \log n_i. \quad \text{(A8)}$$

We note that we do not include $t_i$ on the right-hand side of A8, as most of the person-time at risk comes from other family members, and $t_i$ is relatively unimportant regarding the whole family. However, we run an alternative model

$$\log\{-\log[1 - F_i(t_i)]\} = \log \lambda_0 + \boldsymbol{x}_i'\boldsymbol{\beta} + \log n_i + \gamma \log t_i \quad \text{(A9)}$$

as a robustness check. In A9, $\gamma$ is a parameter to be freely estimated.

# Robustness checks

## 1. Backward model selection

In the main matter, we estimate A4 and A8 by using complementary log-log regression and perform stepwise forward model selection for the SNP covariates. We note that stepwise model selection may stop in a locally optimal model (as opposed to a globally optimal one), and also that it is inconvenient to perform exhaustive search over the whole model space of models 3 and 4 ($2^{33}$= 8,589,934,592 items). Thus, we check the robustness of our results by running stepwise model selection in backward direction. The results are as presented in Table T1.

Regarding HLA class II haplotypes (models 1 and 2), stepwise backward model selection chooses the very same model as forward model selection. Regarding non-HLA SNPs and AID clustering in the index patient (model 3), the results are identical to those of forward model selection.

Regarding non-HLA SNPs and the autoimmune-family phenotype (model 4), backward model selection chooses a slightly larger model than forward model selection (main matter). However, all

the same SNPs that were listed in the main matter are included in the model, and their effects are very similar.

| Table T1. Multivariate models by using stepwise backward model selection.<br>The joint P values concern the HLA class II haplotypes or non-HLA SNPs. | | | |
|---|---|---|---|
| Model 1. Children with multiple autoimmune diseases, total n=1,427, AIC=462, joint P = 0.0019. The AIC of the minimal model (only confounding factors): 470. | | | |
| Covariate | HR | 95 % CI | P value (Wald's test) |
| Female sex | 1.57 | 0.91-2.73 | 0.11 |
| Body mass index (BMI) | 0.91 | 0.8-1.01 | 0.084 |
| Plasma glucose at diagnosis | 0.99 | 0.96-1.01 | 0.34 |
| Blood pH at diagnosis | 4.19 | 0.21-149.7 | 0.39 |
| log-GADA level | 1.02 | 0.87-1.21 | 0.81 |
| log-IA2A level | 0.93 | 0.8-1.1 | 0.42 |
| log-IAA level | 1.09 | 0.88-1.32 | 0.41 |
| log-ICA level | 0.95 | 0.77-1.19 | 0.67 |
| log-ZnT8A level | 0.84 | 0.65-1.08 | 0.19 |
| **DR4-DQ8** | **0.41** | **0.23-0.72** | **0.0019** |
| Model 2. Autoimmune families, total n=842, AIC=852, joint P = 1.00. The AIC of the minimal model (only confounding factors): 852. | | | |
| Covariate | HR | 95 % CI | P value (Wald's test) |
| Female sex | 1.23 | 0.91-1.66 | 0.17 |
| Body mass index (BMI) | 1.03 | 0.97-1.08 | 0.33 |
| **Plasma glucose at diagnosis** | **0.98** | **0.96-1** | **0.023** |
| Blood pH at diagnosis | 3.26 | 0.66-19.02 | 0.17 |

| | | | |
|---|---|---|---|
| log-GADA level | 1.08 | 0.98-1.18 | 0.1 |
| log-IA2A level | 1.03 | 0.94-1.12 | 0.58 |
| log-IAA level | 1.04 | 0.94-1.16 | 0.42 |
| log-ICA level | 0.96 | 0.85-1.09 | 0.56 |
| log-ZnT8A level | 0.91 | 0.8-1.04 | 0.19 |
| Model 3. Children with multiple autoimmune diseases, total n=1,180, AIC=365, joint P = $6.6\times10^{-5}$. The AIC of the minimal model (only confounding factors): 382. | | | |
| Covariate | HR | 95 % CI | P value (Wald's test) |
| Female sex | 1.73 | 0.94-3.23 | 0.082 |
| Body mass index (BMI) | 0.92 | 0.81-1.04 | 0.2 |
| Plasma glucose at diagnosis | 0.96 | 0.93-1 | 0.052 |
| Blood pH at diagnosis | 1.77 | 0.08-73.78 | 0.74 |
| log-GADA level | 0.93 | 0.78-1.12 | 0.46 |
| log-IA2A level | 0.87 | 0.73-1.05 | 0.13 |
| log-IAA level | 1.06 | 0.83-1.32 | 0.63 |
| log-ICA level | 1 | 0.78-1.29 | 0.98 |
| log-ZnT8A level | 0.84 | 0.62-1.11 | 0.24 |
| HLA risk level | 0.78 | 0.59-1.02 | 0.065 |
| **rs2666236 (*NRP1*)** | **0.52** | **0.31-0.83** | **0.0081** |
| rs763361 (*CD226*) | 1.38 | 0.89-2.13 | 0.15 |
| **rs2816316 (*RGS1*)** | **2.17** | **1.2-3.8** | **0.0071** |
| **rs601338 (*FUT2*)** | **1.58** | **1.05-2.4** | **0.028** |
| rs3024505 (*IL10*) | 0.51 | 0.21-1.04 | 0.093 |
| **rs4763879 (*CD69*)** | **1.67** | **1.09-2.55** | **0.019** |

| Model 4. Auotimmune families, total n=691, AIC=709, joint P = 0.003. The AIC of the minimal model (only confounding factors): 716. | | | |
|---|---|---|---|
| Covariate | HR | 95 % CI | P value (Wald's test) |
| Female sex | 1.2 | 0.86-1.66 | 0.28 |
| Body mass index (BMI) | 1.03 | 0.96-1.09 | 0.41 |
| **Plasma glucose at diagnosis** | **0.97** | **0.96-0.99** | **0.0052** |
| Blood pH at diagnosis | 1.47 | 0.27-9.75 | 0.67 |
| **log-GADA level** | **1.11** | **1-1.23** | **0.047** |
| log-IA2A level | 1.02 | 0.92-1.12 | 0.76 |
| log-IAA level | 1.03 | 0.91-1.15 | 0.66 |
| log-ICA level | 0.92 | 0.8-1.06 | 0.24 |
| log-ZnT8A level | 0.93 | 0.81-1.07 | 0.3 |
| HLA risk level | 1.04 | 0.9-1.21 | 0.61 |
| rs6546909 (*DQX1*) | 0.75 | 0.53-1.05 | 0.11 |
| rs7719828 (*LOC645261*) | 1.21 | 0.95-1.54 | 0.12 |
| **rs17696736 (*C12orf30/NAA25*)** | **0.59** | **0.38-0.93** | **0.019** |
| **rs3184504 (*SH2B3*)** | **1.6** | **1.04-2.41** | **0.028** |
| **rs11711054 (*CCR3-CCR5*)** | **0.7** | **0.53-0.92** | **0.011** |
| rs9585056 (*GPR183*) | 1.22 | 0.95-1.55 | 0.11 |

## 2. Bayesian information criterion

The Bayesian information criterion (BIC, Schwarz 1978) is sometimes used as an alternative to AIC. It is based on different statistical principles, and it generally favors a more parsimonious

model than AIC. Thus, in this subsection, we run the stepwise forward model selection to minimize BIC. The results are as presented in Table T2.

In these data, the use of BIC prunes away most SNPs (and HLA class II haplotypes), but DR4-DQ8 and rs11711054 (*CCR3-CCR5*) are still retained in models 1 and 4, respectively. Moreover, their effects are almost equal to those found in the main matter by using AIC. (The differences arise from different sets of other covariates.) The effects of the confounding factors are largely similar to those found in the main matter, plasma glucose level and log-GADA level being the only significant confounders.

**Table T2. Multivariate models by using Bayesian information criterion.**

The joint P values concern the HLA class II haplotypes or non-HLA SNPs.

Model 1. Children with multiple autoimmune diseases, total n=1,427, BIC=462, joint P = 0.0019.

The BIC of the minimal model (only confounding factors): 470.

| Covariate | HR | 95 % CI | P value (Wald's test) |
|---|---|---|---|
| Female sex | 1.57 | 0.91-2.73 | 0.11 |
| Body mass index (BMI) | 0.91 | 0.8-1.01 | 0.084 |
| Plasma glucose at diagnosis | 0.99 | 0.96-1.01 | 0.34 |
| Blood pH at diagnosis | 4.19 | 0.21-149.7 | 0.39 |
| log-GADA level | 1.02 | 0.87-1.21 | 0.81 |
| log-IA2A level | 0.93 | 0.8-1.1 | 0.42 |
| log-IAA level | 1.09 | 0.88-1.32 | 0.41 |
| log-ICA level | 0.95 | 0.77-1.19 | 0.67 |
| log-ZnT8A level | 0.84 | 0.65-1.08 | 0.19 |
| **DR4-DQ8** | **0.41** | **0.23-0.72** | **0.0019** |

| Model 2. Autoimmune families, total n=842, BIC=852, joint P = 1.00. The BIC of the minimal model (only confounding factors): 852. | | | |
|---|---|---|---|
| Covariate | HR | 95 % CI | P value (Wald's test) |
| Female sex | 1.23 | 0.91-1.66 | 0.17 |
| Body mass index (BMI) | 1.03 | 0.97-1.08 | 0.33 |
| **Plasma glucose at diagnosis** | **0.98** | **0.96-1** | **0.023** |
| Blood pH at diagnosis | 3.26 | 0.66-19.02 | 0.17 |
| log-GADA level | 1.08 | 0.98-1.18 | 0.1 |
| log-IA2A level | 1.03 | 0.94-1.12 | 0.58 |
| log-IAA level | 1.04 | 0.94-1.16 | 0.42 |
| log-ICA level | 0.96 | 0.85-1.09 | 0.56 |
| log-ZnT8A level | 0.91 | 0.8-1.04 | 0.19 |
| Model 3. Children with multiple autoimmune diseases, total n=1,180, BIC=382, joint P = 1.00. The BIC of the minimal model (only confounding factors): 382. | | | |
| Covariate | HR | 95 % CI | P value (Wald's test) |
| Female sex | 1.68 | 0.91-3.13 | 0.096 |
| Body mass index (BMI) | 0.92 | 0.81-1.03 | 0.17 |
| Plasma glucose at diagnosis | 0.97 | 0.94-1 | 0.096 |
| Blood pH at diagnosis | 1.64 | 0.07-76.55 | 0.78 |
| log-GADA level | 0.94 | 0.79-1.13 | 0.52 |
| log-IA2A level | 0.9 | 0.76-1.08 | 0.26 |
| log-IAA level | 1.1 | 0.87-1.37 | 0.39 |
| log-ICA level | 0.99 | 0.78-1.26 | 0.92 |
| log-ZnT8A level | 0.84 | 0.63-1.1 | 0.23 |

| | | | |
|---|---|---|---|
| HLA risk level | 0.79 | 0.61-1.04 | 0.089 |
| Model 4. Autoimmune families, total n=691, BIC=712, joint P = 0.0088. The BIC of the minimal model (only confounding factors): 716. | | | |
| Covariate | HR | 95 % CI | P value (Wald's test) |
| Female sex | 1.19 | 0.85-1.65 | 0.3 |
| Body mass index (BMI) | 1.03 | 0.97-1.09 | 0.3 |
| **Plasma glucose at diagnosis** | **0.98** | **0.96-0.99** | **0.0097** |
| Blood pH at diagnosis | 1.92 | 0.34-12.89 | 0.48 |
| **log-GADA level** | **1.11** | **1-1.23** | **0.043** |
| log-IA2A level | 1.01 | 0.91-1.11 | 0.9 |
| log-IAA level | 1.02 | 0.91-1.15 | 0.68 |
| log-ICA level | 0.94 | 0.81-1.08 | 0.37 |
| log-ZnT8A level | 0.94 | 0.82-1.08 | 0.43 |
| HLA risk level | 1.03 | 0.89-1.2 | 0.72 |
| **rs11711054 (*CCR3-CCR5*)** | **0.7** | **0.53-0.92** | **0.011** |

## 3. Alternative person-time

The models of the main matter are based on equations A4 and A8. The derivation of A4 assumes that the baseline risk is constant in time, and A8 is based on mainly ad-hoc arguments. Consequently, it is possible that the relationship between the person-time and the outcome of interest is misspecified in both models. As the person-time at risk affects the probability of a positive outcome, it is worthwhile to further investigate this issue.

As a robustness check, we run estimation and model choice by using alternative specifications for the person-time (equations A5 and A9). For these analyses, we use AIC and stepwise forward model selection. The results are as presented in Table T3.

Regarding HLA class II haplotypes (models 1 and 2), model specifications A5 and A9 give the very same model, as A4 and A8 (main matter), notwithstanding the log-age covariate. In model 3 (non-HLA SNPs and AID clustering within the child), rs763361 (*CD226*) has been left out of the model, but the effects of the other SNPs have the same directions and a similar pattern of significance.

For model 4 (non-HLA SNPs and autoimmune families), the results are very similar to those given in the main matter. The same set of SNPs has been chosen by using both specifications (A8 in the main matter and A9 here).

**Table T3. Multivariate models by using alternative specifications of person-time.**

The joint P values concern the HLA class II haplotypes or non-HLA SNPs.

Model 1. Children with multiple autoimmune diseases, total n=1,427, AIC=459, joint P = 0.0018.

The AIC of the minimal model (only confounding factors): 470.

| Covariate | HR | 95 % CI | P value (Wald's test) |
|---|---|---|---|
| Log-age | 1.4 | 0.83-2.43 | 0.22 |
| Female sex | 1.55 | 0.9-2.69 | 0.11 |
| Body mass index (BMI) | 0.94 | 0.83-1.04 | 0.27 |
| Plasma glucose at diagnosis | 0.99 | 0.96-1.02 | 0.41 |
| Blood pH at diagnosis | 3.09 | 0.17-103.51 | 0.49 |
| log-GADA level | 1.06 | 0.89-1.25 | 0.53 |
| log-IA2A level | 0.95 | 0.81-1.12 | 0.55 |
| log-IAA level | 0.98 | 0.77-1.22 | 0.85 |
| log-ICA level | 0.93 | 0.75-1.16 | 0.53 |

| | | | |
|---|---|---|---|
| log-ZnT8A level | 0.86 | 0.66-1.1 | 0.24 |
| **DR4-DQ8** | **0.41** | **0.23-0.71** | **0.0018** |
| Model 2. Autoimmune families, total n=842, AIC=852, joint P = 1.00. The AIC of the minimal model (only confounding factors): 852. | | | |
| Covariate | HR | 95 % CI | P value (Wald's test) |
| Log-age | 0.82 | 0.63-1.08 | 0.16 |
| Female sex | 1.23 | 0.91-1.66 | 0.17 |
| Body mass index (BMI) | 1.04 | 0.98-1.09 | 0.2 |
| **Plasma glucose at diagnosis** | **0.98** | **0.96-1** | **0.033** |
| Blood pH at diagnosis | 3.1 | 0.64-17.92 | 0.19 |
| log-GADA level | 1.09 | 0.99-1.19 | 0.067 |
| log-IA2A level | 1.04 | 0.95-1.14 | 0.44 |
| log-IAA level | 1 | 0.88-1.13 | 0.98 |
| log-ICA level | 0.96 | 0.84-1.09 | 0.48 |
| log-ZnT8A level | 0.92 | 0.8-1.05 | 0.22 |
| Model 3. Children with multiple autoimmune diseases, total n=1,180, AIC=364, joint P = 0.00018. The AIC of the minimal model (only confounding factors): 382. | | | |
| Covariate | HR | 95 % CI | P value (Wald's test) |
| Log-age | 1.46 | 0.8-2.8 | 0.23 |
| Female sex | 1.73 | 0.94-3.24 | 0.08 |
| Body mass index (BMI) | 0.95 | 0.83-1.07 | 0.4 |
| Plasma glucose at diagnosis | 0.97 | 0.93-1 | 0.066 |
| Blood pH at diagnosis | 1.4 | 0.07-58.41 | 0.84 |
| log-GADA level | 0.95 | 0.79-1.14 | 0.59 |

| | | | |
|---|---|---|---|
| log-IA2A level | 0.89 | 0.74-1.06 | 0.19 |
| log-IAA level | 0.96 | 0.73-1.24 | 0.79 |
| log-ICA level | 0.99 | 0.77-1.27 | 0.95 |
| log-ZnT8A level | 0.87 | 0.64-1.14 | 0.33 |
| HLA risk level | 0.77 | 0.59-1.02 | 0.058 |
| **rs2666236 (*NRP1*)** | **0.53** | **0.32-0.85** | **0.0099** |
| **rs2816316 (*RGS1*)** | **2.14** | **1.17-3.75** | **0.0091** |
| **rs4763879 (*CD69*)** | **1.65** | **1.08-2.53** | **0.02** |
| **rs601338 (*FUT2*)** | **1.56** | **1.03-2.36** | **0.033** |
| rs3024505 (*IL10*) | 0.53 | 0.22-1.1 | 0.12 |
| Model 4. Autoimmune families, total n=691, AIC=711, joint P = 0.0064. The AIC of the minimal model (only confounding factors): 716. | | | |
| Covariate | HR | 95 % CI | P value (Wald's test) |
| Log-age | 0.84 | 0.62-1.16 | 0.29 |
| Female sex | 1.21 | 0.87-1.68 | 0.25 |
| Body mass index (BMI) | 1.03 | 0.97-1.1 | 0.31 |
| **Plasma glucose at diagnosis** | **0.97** | **0.96-0.99** | **0.0069** |
| Blood pH at diagnosis | 1.51 | 0.28-9.95 | 0.65 |
| **log-GADA level** | **1.11** | **1.01-1.24** | **0.035** |
| log-IA2A level | 1.02 | 0.92-1.13 | 0.69 |
| log-IAA level | 0.98 | 0.86-1.12 | 0.82 |
| log-ICA level | 0.92 | 0.8-1.06 | 0.23 |
| log-ZnT8A level | 0.95 | 0.82-1.09 | 0.46 |
| HLA risk level | 1.03 | 0.88-1.2 | 0.73 |

| | | | |
|---|---|---|---|
| **rs11711054 (*CCR3-CCR5*)** | **0.71** | **0.53-0.92** | **0.013** |
| rs7719828 (*LOC645261*) | 1.22 | 0.96-1.55 | 0.1 |
| rs6546909 (*DQX1*) | 0.76 | 0.53-1.05 | 0.11 |
| rs9585056 (*GPR183*) | 1.2 | 0.94-1.53 | 0.14 |

## 4. Conclusion

The results are largely insensitive to direction of model choice (robustness check 1), albeit backward model selection chooses a few more non-HLA SNPs for autoimmune families (model 4). The results are somewhat sensitive towards using BIC in place of AIC as a model choice criterion (robustness check 2). However, the results we present in the main matter are not only justified by AIC, but also by univariate and multivariate significance tests (Wald and LR, respectively). Moreover, the effects of DR4-DQ8 and CCR5 are still retained, if BIC is used in place of AIC. The results are insensitive towards using log-age as a covariate, and thus altering the specification of person-time (robustness check 3). In this case, the results differ only regarding one SNP (rs763361) in model 3.

# References

McCullagh P, Nelder J.  Generalized linear models. 2nd ed. Boca Raton: Chapman and Hall/CRC; 1998.

Schwarz G E : Estimating the dimension of a model. Ann Stat. 1978;6: 461–464.
